# Prolonged FGF signaling is necessary for lung and liver induction in *Xenopus*

**DOI:** 10.1186/1471-213X-12-27

**Published:** 2012-09-18

**Authors:** Emily T Shifley, Alan P Kenny, Scott A Rankin, Aaron M Zorn

**Affiliations:** 1 Perinatal Institute, Divisions of Developmental Biology, University of Cincinnati, Cincinnati, OH, 45229, USA; 2 Divisions of Neonatology Cincinnati Children's Research Foundation and Department of Pediatrics College of Medicine, University of Cincinnati, Cincinnati, OH, 45229, USA

**Keywords:** FGF, ERK, AKT, Lung, Liver, Foregut, Endoderm, Organ induction, *Xenopus*

## Abstract

**Background:**

FGF signaling plays numerous roles during organogenesis of the embryonic gut tube. Mouse explant studies suggest that different thresholds of FGF signaling from the cardiogenic mesoderm induce lung, liver, and pancreas lineages from the ventral foregut progenitor cells. The mechanisms that regulate FGF dose *in vivo* are unknown. Here we use *Xenopus* embryos to examine the hypothesis that a prolonged duration of FGF signaling from the mesoderm is required to induce foregut organs.

**Results:**

We show that both mesoderm and FGF signaling are required for liver and lung development in *Xenopus;* formally demonstrating that this important step in organ induction is conserved with other vertebrate species. Prolonged contact with the mesoderm and persistent FGF signaling through both MEK and PI3K over an extended period of time are required for liver and lung specification. Inhibition of FGF signaling results in reduced liver and lung development, with a modest expansion of the pancreas/duodenum progenitor domain. Hyper-activation of FGF signaling has the opposite effect expanding liver and lung gene expression and repressing pancreatic markers. We show that FGF signaling is cell autonomously required in the endoderm and that a dominant negative FGF receptor decreases the ability of ventral foregut progenitor cells to contribute to the lung and liver buds.

**Conclusions:**

These results suggest that the liver and lungs are specified at progressively later times in development requiring mesoderm contact for different lengths of time. Our data suggest that this is achieved at least in part through prolonged FGF signaling. In addition to providing a foundation for further mechanistic studies on foregut organogenesis using the experimental advantages of the *Xenopus* system, these data have implications for the directed differentiation of stem cells into foregut lineages.

## Background

In the Fibroblast Growth Factor (FGF) signaling pathway, secreted ligands bind to transmembrane tyrosine kinase FGF receptors causing dimerization and activation of a number of intracellular signal transduction cascades including the mitogen-activated protein kinase (MEK) and phosphoinositide 3-kinase (PI3K), which phosphorylate Erk and Akt, respectively [1]. FGF signals regulate cellular differentiation, proliferation, and survival in many contexts and studies in mice, chick, and zebrafish have shown that FGF mediated mesenchymal-epithelial interactions play numerous roles in the developing gut tube [2-4]. During gastrulation, FGF signaling patterns the primitive gut tube by promoting posterior over anterior cell fate in the endoderm [5]. Then only hours later, FGF signals from the anterior lateral plate and cardiac mesoderm segregate the pancreas, liver, and lung lineages from a pool of ventral foregut progenitor cells [6-12]. Recent studies in zebrafish suggest that FGF signaling acts in part by restricting hepatic competence of the endoderm along the anterior-posterior (A-P) axis [13,14]. Additionally, FGFs are important for the outgrowth and morphogenesis of many organ buds during fetal development; for instance mesenchymal FGF10 controls lung branching [15,16], pancreas proliferation and growth [17,18], stomach morphogenesis [19], and hepatopancreatic fate [20]. Considering these multiple context-dependent activities, it is likely that a better understanding of the precise temporal roles of FGF signaling during endoderm organogenesis will inform approaches to direct the differentiation of human stem cells *in vitro*[2,21].

In this study we investigated the role that FGF signaling plays in the specification of foregut organs in *Xenopus* embryos. In zebrafish and chick, FGF signals (along with BMP and Wnt) have been shown to be essential for hepatic specification [6,7]. Additionally, *in vitro* studies using mouse embryo foregut explant cultures from 0–7 somite-stages (ss) of development have suggested that FGF signals from the cardiac and lateral plate mesoderm regulate the induction of the pancreas, liver, and lungs in a dose-dependent manner [8,10]. Little or no FGF signaling is required for ventral endodermal explants from early somite-stage mouse embryos to turn on the pancreas marker *Pdx1*, whereas explants cultured with cardiac mesoderm or recombinant FGF2 express the liver marker *Albumin*[12] and higher FGF doses stimulate expression of the thyroid/lung marker *Nkx2.1*[8]. Similar dose-responsive FGF effects have been observed during the differentiation of human ES cells to foregut lineages [22,23]. Downstream of FGF receptor signaling, it has been shown that in mouse embryos the MEK branch of the FGF pathway is necessary for liver *Albumin* and *Alpha-fetoprotein* expression, while the PI3K branch promotes hepatoblast proliferation [9]. These data have led to a model of foregut organ development where different doses of FGF specify the different foregut lineages: very low or absent FGF levels are required for pancreas, intermediate FGF levels promote liver, and high FGF levels are required for lung. The mechanisms by which different thresholds of FGF are achieved *in vivo* are unknown, in part because mouse embryos are difficult to manipulate at these early stages in development.

*Xenopus* embryos have increasingly proven to be a valuable model to study the mechanisms of organogenesis [24,25]. Horb and Slack have shown that signals from the mesoderm between stages NF15 (0 ss) to NF42 (organ bud stage) are required for *Xenopus* endodermal explants to become regionally specified into anterior and posterior organ lineages [26]. Consistent with a conserved role for FGF signaling in *Xenopus* foregut organ induction, multiple FGF ligands and receptors are expressed in the *Xenopus* foregut mesenchyme and endoderm between stages NF15-42 [27] . Moreover, FGF signaling is necessary for the induction of liver gene expression in cultured ectodermal explants *in vitro*[28] and Akt signaling is required for later pancreas and stomach progenitor cell survival [29].

Despite these suggestive data, the role of FGF signaling in *Xenopus* foregut organ specification *in vivo* has not formally been determined. In this study, we show that: 

1. The pancreas, liver and lung are specified at progressively later times in *Xenopus* development, and that the liver and lungs require progressively longer mesoderm contact;

2. FGF signaling is required for lung and liver specification *in vivo* and this is, at least in part, a cell autonomous requirement in the endoderm;

3. Foregut endoderm in which FGF signaling is experimentally blocked appears to remain in a progenitor-like state;

4. Ectopic activation of the FGF pathway expands liver and lung development, and represses pancreatic fate;

5. The high levels of FGF necessary for lung and liver induction appear to be achieved through FGF signaling over an extended period of time via both the MEK and PI3K pathways.

This temporal requirement for FGF signaling in foregut lineage segregation provides the foundation for future mechanistic studies in *Xenopus*, and may impact studies aimed at inducing different foregut lineages from pluripotent stem cells.

## Methods

### Embryo manipulations

**Figure 1:**
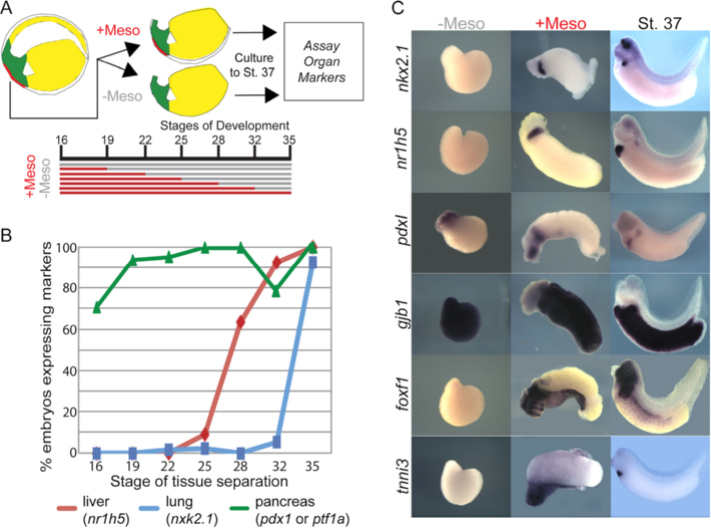
**Foregut organ specification requires prolonged contact with the cardiac-lateral plate mesoderm.** (**A**) Diagram of the experimental design. Ventral explants were cultured with (+Meso) or without mesoderm (−Meso) during the indicated time points and assayed by *in situ* hybridization at stage NF37. (**B**) Summary of experimental results showing the percentage of explants with mesoderm removed at different times expressing liver (*nr1h5*), lung/thyroid (*nkx2.1*) and pancreas (*pdx1* or *ptf1a*) markers at stage NF37, n > 20 explants for each condition and each probe. (**C**) Representative explants cultured from stage NF16 to NF37 with or without mesoderm and corresponding whole embryo controls assayed by *in situ* hybridization with the indicated markers. Endodermal (*gjb1*) or mesodermal (*foxf1* and *tnni3*) specific markers demonstrated clean separation of the tissues.

*Xenopus laevis* embryos were cultured as previously described [30]. All animal experiments complied with the “Animal Research: Reporting *in vivo* Experiments” (ARRIVE) guidelines and were approved by the Cincinnati Children’s Research Foundation IACUC committee (protocol #0B12097). For microdissection, ventral explants were dissected from embryos using eyelash blades and hair loops. At the indicated developmental time half of the explants had the mesoderm separated using hair loops in 5 units/ml Dispase (BD Biosciences) for 10–15 min (Figure 1A). Endoderm explants (+meso and –meso) were then cultured in 0.5xMBS to Stage 37 for analysis. Synthetic RNA for microinjection was transcribed using the mMessage Kits (Ambion) and purified on Microspin-6 columns (BioRad). The following plasmids were used for mRNA synthesis (enzymes used to linearize DNA templates, RNA polymerase): pRN3 GFP (SfiI, T3); pCS2 + β-gal (NotI, Sp6); dnFGFR (pxFD/XSS) (EcoRI, SP6); caFGFR (iFGFR1) (Not1, SP6). Embryos with clear dorsal-ventral pigment were selected for injections into the bottom of dorsal-vegetal blastomeres at the 4-8-cell stage to target large regions of the foregut mesendoderm and into the D2.1 cells at the 16-cell stage to target foregut endoderm avoiding the mesoderm. Lineage labels were used to confirm the correct targeting. Cell soluble inhibitors were dissolved in DMSO and added to the media at the following working concentrations: PD173074 (300 μM; TOCRIS), U0126 (Cell Signaling, 300 μM), LY294003 (Cell Signaling, 40 μM), and SU5402 (10 μM with 0.1 M ATP). B/B Homodimerizer (Clontech) was dissolved in 100% ethanol and used at 1.25 μM working concentration. Explants were cultured in 100 ng/ml of human recombinant FGF2 (Invitrogen) in 0.5XMBS + 0.1%BSA. 

### *In situ* hybridization

*In situ* hybridizations were performed as previously described [31] using the following probes: *nr1h5* (formally *for1*, Xenbase.org) [32], *pdx1*[33], *ptf1α*[34], *nkx2.1*[35], *tnni3* (formally *cardiac-troponin*, Xenbase.org) [36], *foxf1*[37], *gjb1*[38], *hhex*[39], *nkx2.5*[40], *hnf4α*[41]. Image-J software was used to measure the average size of the *hhex* and *pdx1* expression domains +/− S.D. in injected and control sibling embryos.

### Immunostaining and Western blot analysis

For Western blots, five embryos per sample were lysed in a TLB buffer (1% Triton X 100, 25 mM Tris pH 7.4, 150 mM NaCl, 2 mM Na_3_VO_4_, 2.5 mM NaF and 25 mM B-glycerophosphate) with protease and phosphatase inhibitors each diluted 1:100: phosphatase inhibitor cocktail II (Sigma), PhosSTOP (Roche), protease inhibitor cocktail (Sigma). Samples were run on a 10% polyacrylamide gel and transferred to an Immobilon membrane (Millipore). The membrane was incubated with the following antibodies: mouse anti-dpErk1/2 (1:250, Sigma), rabbit anti-Erk2 (1:1000, Cell Signaling), rabbit anti-pAkt (1:1000, Cell Signaling), and rabbit anti-AKT (1:1000, Cell Signaling) and analyzed using the ECL Plus system (GE Healthcare) and a FUJIFILM LAS-4000 luminescent analyzer.

For immunofluorescence, *Xenopus* embryos were fixed in MEMFA for 2 h at RT, bisected with a razor blade, and stored in Dent’s fixative (80%MetOH + 20%DMSO). For confocal analysis, the embryo pieces were rehydrated though a methanol series, blocked with BBT (PBS, 1%bovine albumin, 0.1%Triton) for 2 h and BBT with 5% serum for 1 h, incubated with primary antibody overnight at 4°, washed in PBS 0.2%Triton, incubated with secondary antibody overnight at 4°, washed again, dehydrated in methanol, then cleared with a Benzyl Benzoate and Benzyl Alcohol mix (2:1). For immunofluorescence, the following antibodies were used, primary: rabbit anti-dpErk1/2 (1:300, Cell Signaling), mouse anti-GFP (1:300, Clonetech), rabbit anti-phospho-Histone H3 (1:300, Cell Signaling), and rabbit anti-active-Caspase 3 (1:300, Cell Signaling); and secondary antibodies: anti-rabbit-CY5 (1:500, Jackson), anti-mouse-CY2 (1:500, Jackson) or goat anti-rabbit-AP (1:5000, Jackson).

## Results

### Pancreas, liver, and lung are specified at progressively later times in development through prolonged interactions with cardiac-lateral plate mesoderm

As a first step in characterizing the potential role of FGFs in *Xenopus* foregut organ induction we carefully examined when during development different foregut lineages were specified. Horb and Slack have previously demonstrated in *Xenopus* that mesodermal signals between stages NF15 to NF42 are required in order for the endoderm to become regionally specified and express pancreas, liver, and intestinal markers at stage NF42 [26]. In order to determine whether distinct lineages are specified at different times, we performed a series of microdissection experiments isolating ventral explants and removing the ventral cardiac/lateral plate mesoderm from the endoderm at different times in development (Figure 1A). Explants were cultured until stage NF37 (~40 ss) and analyzed by *in situ* hybridization for expression of early lineage markers of the pancreas (*pdx1* and *pft1a*; [33,42]), liver (*nr1h5*; [32]) and lung/thyroid (*nkx2.1*; [35]) (Figure 1B, C). As controls to verify effective separation of the endoderm from mesoderm tissue, we examined the expression of the pan-endodermal marker *gjb1*[38], the lateral plate mesoderm marker *foxf1*[37], and the cardiac mesoderm marker *tnni3*[36] (Figure 1C). These controls demonstrated that our method of removing the mesoderm by dispase treatment and manual pealing off the tissue with hairloops effectively produced endoderm explants without *foxf1*+ and *tnni3*+ mesoderm.

These experiments showed that the expression of early pancreas, liver, or lung markers in the endoderm required mesoderm contact for different periods of time. Interestingly, we observed the pancreas-duodenum marker *pdx1* was expressed in explants >75% of the time regardless of when the mesoderm was removed between stages NF16-35 (Figure 1B,C). We obtained similar results with another pancreas marker *ptf1a*, suggesting that as early as stage NF16 the endoderm has received sufficient signals to activate expression of pancreatic progenitor markers by stage NF35. In contrast, expression of the lung and liver markers required longer durations of mesodermal contact. Expression of the liver marker *nr1h5* required mesoderm contact until stages NF25-28, after which point the mesoderm was no longer required (Figure 1B,C). In contrast, *nkx2.1* expression was not induced in endoderm explants unless mesoderm was kept in contact throughout stage NF35 (36 ss; Figure 1B,C). In explants cultured with mesoderm through stage NF35, the *nkx2.1*+ tissue was observed in two discreet domains immediately dorsal-posterior to the heart, indicative of lung tissue. We conclude that the pancreas, liver, and lungs are specified at progressively later times in development in a caudal-to-rostral progression along the A-P axis. The most caudal tissue the pancreas is specified first, followed by liver, which requires mesoderm contact until NF31 and then the most rostral organ the lung is specified last requiring mesoderm contact up to NF35.

### FGF signaling is active in the *Xenopus* foregut endoderm during organ induction

**Figure 2:**
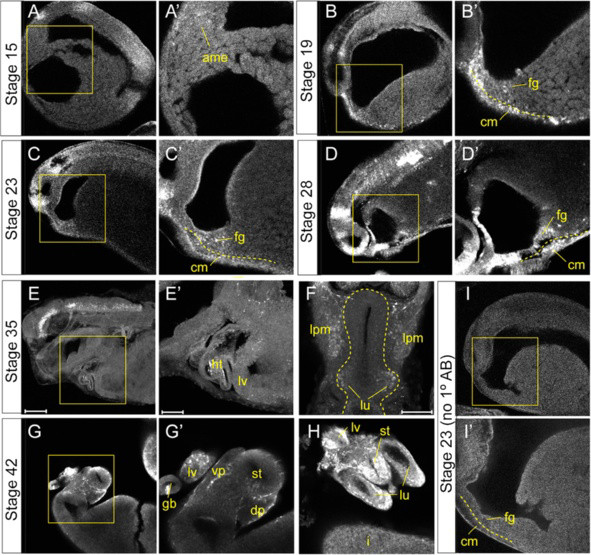
**FGF signaling is active in the foregut endoderm during organ induction.** Confocal immunostaining of bisected *Xenopus* embryos show active FGF-MEK signaling with di-phospho ERK1/2 (pErk) (white) in the developing foregut tissue at the indicated stages (anterior left, ventral down). Images are 10X magnification with independent scans of the boxed regions at 20X magnification. (**A**) Stage NF15 embryos show a low level of FGF signaling in the migrating anterior mesendoderm (ame). (**B**) Stage NF19, (**C**) NF23 and (**D**) NF28 show pErk staining in the ventral foregut endoderm (fg) as well as the underlying cardiac mesoderm (cm). The dashed yellow line indicates the boundary between the endoderm and mesoderm. (**E**) Mid-sagittal and (**F**) transverse optical sections of stage NF35 embryos show pErk staining in the liver epithelium (lv), heart (ht) and nascent lung buds (lu) and lateral plate mesoderm (lpm). (**G** and **H**) Stage NF42 gut tubes show pErk in liver bud (lv), gal bladder (gb), dorsal (dp) and ventral pancreatic buds (vp), the stomach (st) and the distal tips of the lung buds (lu). (**I**) Stage NF23 control embryos with no primary antibody.

Our tissue separation experiments show that complete organ induction requires mesodermal contact between stages NF16-35. A survey of the literature indicates that many FGF ligands (FGF1-4,6,8,9,14,19,22,23) and receptors are expressed in the *Xenopus* foregut region during this time in development [27,43]. To investigate if and when the *Xenopus* ventral foregut endoderm is responding to FGF signaling we examined di-phosphorylated Erk1/2 (pErk) immunostaining as a read-out of active FGF/MEK signaling. In the gastrula embryo, pErk was not detected in the endoderm and was restricted to the involuting mesoderm, as previously described [44]. We first detected a low level of pErk in the anterior mesendoderm at stage NF15 as it migrates to its final position in the ventral foregut (Figure 2A). Between stages NF19-28 robust pErk was present in the ventral foregut progenitors and in the adjacent cardiac and lateral plate mesoderm (Figure 2B-D). By stage NF35 when lung and liver specification markers begin to be expressed pErk is detected in the thickening hepatic epithelium and the nascent lung buds, as well as in the heart and lateral plate mesoderm (Figure 2E, F). In stage NF42 gut tubes, we detected pErk in the liver and pancreatic buds, stomach, and the distal tips of the lung buds consistent with a role for FGF signaling during organ bud outgrowth (Figure 2G, H). This data reveals that between stages NF15-35, the period when mesoderm is required for liver and lung specification, the endoderm is experiencing active pErk signaling. 

### FGF signaling induces lung and liver, and represses early pancreas fate

**Figure 3:**
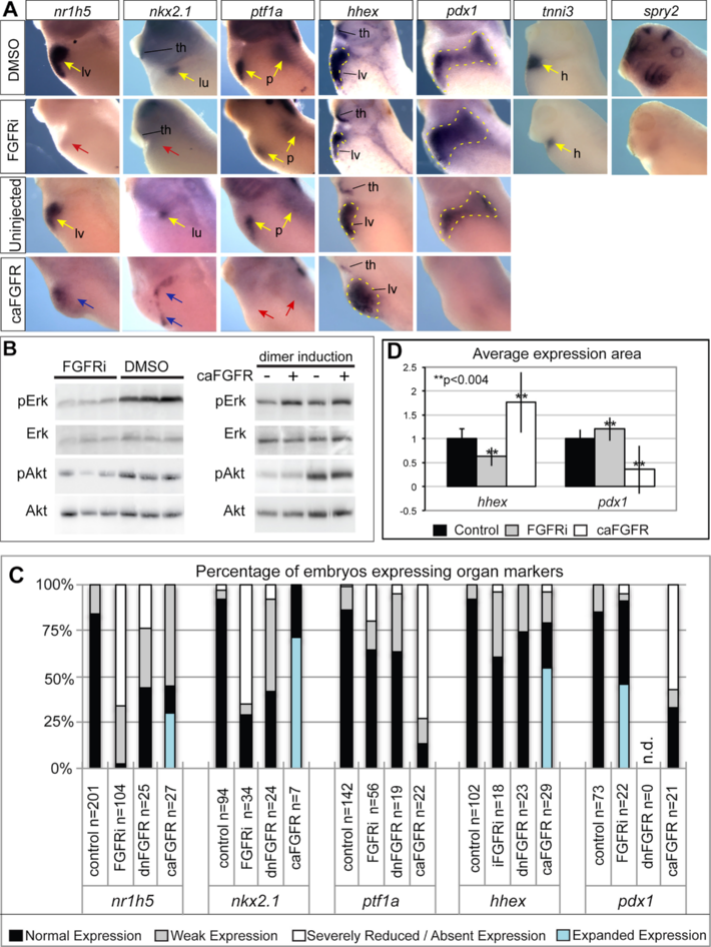
**FGF signaling is required for lung and liver specification at the expense of pancreas**. (**A**) *In situ* hybridization with the indicated probes in control, FGF-inhibited or FGF-activated embryos. Embryos were cultured in DMSO or PD173074 (FGFRi) from NF18 to NF35 to inhibit FGF signaling. Control uninjected or embryos injected with RNA encoding an inducible FGF receptor (caFGFR; 20 pg) into vegetal blastomeres at the 4/8-cell stage, were treated with the homodimerizing drug from NF18 to NF35 to activate FGF signaling. Yellow arrows indicate normal expression, red arrows absent expression, blue arrows ectopic expression; yellow dash outlines expression boundaries. p; pancreas, lv; liver, lu; lung, th; thyroid, h; heart. (**B**) Western blot analysis of pErk, total Erk, pAkt and total Akt in embryos cultured in either PD173074 (FGFRi) or DMSO from stage NF18 to NF35 (in triplicate). Western blot analysis of embryos injected with caFGFR(+) and treated from NF18 to NF35 with B/B Homodimerizer show increased FGF/pErk signaling compared to uninjected controls at Stage NF 23 (lanes1 + 2) and Stage NF 35 (lanes3 + 4). (**C**) Summary of gene expression in FGF-inhibited (FGFRi or dnFGFR; 3 ng) and FGF-activated (caFGFR; 20 pg) embryos. Controls include DMSO treated, β-gal injected and uninjected B/B dimerizing drug treated, none of which had an obvious impact on gene expression. (**D**) Quantification of average *hhex* and *pdx1* expression areas in control and FGF-manipulated embryos. ImageJ software was used to measure the expression area with the average expression area of controls set to 1. Averages are based on n > 13 embryos for each condition and marker from at least two independent experiments, standard deviation and significance based on *t*-test (**p < 0.004).

Multiple FGF ligands and receptors are expressed in the *Xenopus* foregut during organ induction [27,43]. In order to inhibit all FGF signaling in the foregut endoderm, we cultured embryos from stage NF18 to NF35 with a small molecule inhibitor PD173074 (FGFRi), which blocks FGF receptor activity [45] and analyzed them for mature liver (*nr1h5*), pancreas (ptf1α), lung (*nkx2.1*), and heart (*tnni3*) markers (Figure 3A). Western blot analysis confirmed that FGFRi treatment dramatically reduced FGF/pErk and FGF/pAkt activity (Figure 3B) and blocked expression of the FGF target gene *spry2* (Figure 3A). FGFRi treatment resulted in a dramatic reduction or complete loss of liver (97% of embryos, n = 104) and lung (71%, n = 34) marker expression (Figure 3 A,C). We did not observe any obvious impact on *nkx2.1* expression in the thyroid region. This FGF requirement in lung development is similar to that recently described by Wang *et al.*[46]. In contrast, expression of the early pancreas marker ptf1α was only reduced in 37% (n = 56) of the FGF-inhibited embryos, suggesting that pancreas specification requires little if any FGF signaling during these stages (Figure 3 A,C). Treatment with a second independent FGF receptor inhibitor SU5402, or injection of RNA encoding a dominant negative FGF receptor (dnFGFR) into the presumptive foregut mesendoderm at the 4–8 cell stage [47] caused a similar loss of liver and lung gene expression at stage NF35, with little if any impact on the pancreas gene expression relative to controls (Figure 3C, data not shown). 

To test whether FGF signaling was sufficient to induce lung and liver fate we cultured foregut endoderm explants lacking mesoderm with recombinant FGF2, but this was not sufficient to induce *nr1h5* or *nkx2.1* expression (Additional file 1: Figure S1). This suggests that other mesodermal signals in additional to FGFs are also required, the most likely candidate being BMPs, which we have recently shown is also required to maintain foregut progenitors [48]. To overcome this complication, we injected the presumptive foregut mesendoderm with a drug inducible FGFR1 construct (caFGFR) [49]. When injected embryos were treated from stage NF18 to NF35 with the drug “B/B-Homodimerizer” (Clontech) this causes receptor clustering and ligand-independent FGFR signaling as measured by increased pErk levels (Figure 3B). Activation of the caFGFR caused an expansion of liver (*nr1h5*) and lung (*nkx2.1*) expression domains in 30% and 75% of the embryos respectively (Figure 3A,C). In contrast, the pancreas marker *ptf1α* was reduced or absent in 86% of the activated caFGFR embryos (Figure 3A,C).

We next wanted to determine what became of the presumptive liver and lung foregut endoderm when FGF signaling was blocked. One possibility was that it adopted a more posterior fate, but analysis of FGFRi embryos with intestinal markers indicated that this was not the case (data not shown). We next tested whether FGF signaling was required to maintain early foregut progenitor identity prior to lineage segregation. We therefore examined the expression of *hhex*, which is initially expressed throughout the foregut endoderm, but then becomes restricted to the liver and thyroid as organ induction proceeds [39]. However, in embryos treated with the FGFRi from stage NF15 to NF23, or embryos injected with dnFGFR RNA we found that *hhex* expression was largely unaffected at stage NF23 (Additional file 2: Figure S2A), indicating that FGF signaling is not required to maintain the foregut progenitors at this stage. In addition when we examined *hhex* expression in embryos treated with FGFRi from NF18-35; *hhex* was still present in most embryos, although the size of the expression domain was significantly reduced (Figure 3A,C,D). This result was in contrast to the liver specification marker *nr1h5*, which was absent in most FGFRi embryos, suggesting that when FGF signaling is inhibited the foregut cells are blocked in a progenitor-like state.

We also closely examined *pdx1* expression because mouse explant experiments suggest that in the absence of cardiac mesoderm or absence of FGF, the foregut adopts a *pdx1*+ pancreatic/duodenal fate rather than liver fate [12]. In *Xenopus pdx1* is expressed in the pancreatic/duodenal progenitors prior to the expression of *ptf1a*. FGFRi treatment caused a modest, but significant expansion in the size of the *pdx1*+ expression domain (Figure 3A,C,D). Examination of *hhex* and *pdx1* in embryos where FGF signaling was ectopically activated by the caFGFR revealed the opposite phenotype with a significant expansion of *hhex* and a reduced or absent *pdx1* expression domain (Figure 3A,C,D).

Together these results indicate that FGF signaling is required for hepatic specification from *hhex* + progenitors. The data further suggests that similar to what has been reported in mice, FGF regulates the choice of *Xenopus* endoderm progenitors cells to adopt liver versus pancreas fates.

### FGF signaling is required autonomously in the endoderm

The *Xenopus* lateral plate and pre-cardiac mesoderm is also patterned by FGF signaling [50-52]. Since the cardiac mesoderm, in particular, is implicated in foregut organ induction it was possible that the effects on endoderm gene expression we observed in FGF-inhibited embryos were secondary to mesodermal changes. We therefore examined the expression of the cardiac progenitor marker *nkx2.5* at stage NF23 and the myocardial differentiation marker *tnni3* at stage NF35. Although *nkx2.5* and *tnni3* expression were reduced in approximately 50% of FGF-inhibited embryos, both heart markers were almost always present (Figure 3A; Additional file 2: Figures S2A and Additional file 3: Figure S3A). This suggested that impaired cardiac development was unlikely to completely explain the loss of liver and lungs.

**Figure 4:**
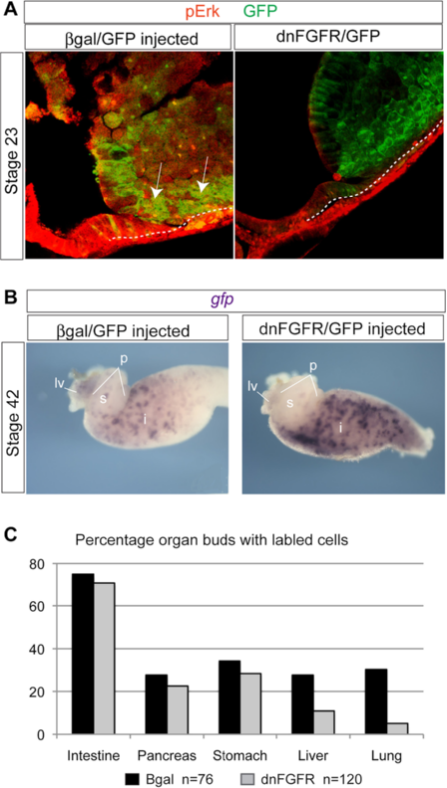
**Inhibition of FGF signaling in *Xenopus* embryos.** (**A**) Confocal immunostaining of pErk (red) and a GFP lineage tracer (green) in the foregut region of bisected stage NF23 *Xenopus* embryos that were injected into the presumptive foregut endoderm cells at the 16-cell stage with RNA encoding either β-gal (3 ng) and GFP or dnFGFR (3 ng) and GFP. The β-gal/GFP injected embryos show pErk in GPF + cells whereas in dnFGFR/GFP injected embryos pErk is undetectable in GFP + foregut cells. (**B**) A representative stage NF42 gut tube from βgal/GFP and dnFGFR/GFP injected embryos assayed by *in situ* hybridization for *gfp* RNA to show mosaic contribution of labeled cells to different organ buds. p; pancreas, lv; liver, s; stomach, i; intestine. (**C**) Summary showing percentage of organ buds containing labeled cells indicates a decreased contribution to the lung and liver buds in dnFGFR injected embryos (n = 120) compared to βgal controls (n = 70).

To directly test the cell-autonomous need for FGF signaling in foregut endoderm progenitors, we injected RNA encoding GFP along with dnFGFR or β-galactosidase (β-gal) RNA into the D2.1 blastomeres at the 16-cell stage, which targets the RNA to the foregut endoderm, and avoids the mesoderm. Injection of the dnFGFR into the mesoderm frequently causes gastrulation defects [47] and we observed this in about 50% of our 4-cell stage injections in Figure 3. In contrast the foregut-targeted embryos gastrulated normally and the lineage label showed that the anterior mesendoderm migrated correctly to the ventral foregut position (Figure 4A). Immunostaining of injected embryos confirmed that dnFGFR/GFP expressing cells lack robust pErk activity at stage NF23 (Figure 4A). We then scored isolated gut tubes at stage NF42 to determine which tissues the lineage labeled cells had contributed. 

The most severe phenotype in these experiments was a complete loss of all discernable foregut organ buds, which never occurred in controls (data not shown); consistent with foregut endoderm requiring FGF signaling to induce organ lineages. In gut tubes with mosaic expression of labeled cells, we found that control β-gal/GFP cells contributed to the liver in 28% and the lung in approximately 30% of embryos, whereas dnFGFR/GFP-expressing cells only contributed to the liver in 11% and the lung in 5% of embryos (Figure 4B, C). Importantly, *gfp* positive cells were not detected in the cardiac mesoderm, verifying that the RNA was specifically targeted to the endoderm. This excludes the possibility that the defects in liver and lung contribution were only secondary to cardiac defects and demonstrates a cell-autonomous requirement for FGF signaling in the endoderm. There was little difference in contribution of control βgal or dnFGFR expressing cells to the intestine, pancreatic buds, or stomach (Figure 4B, C), suggesting that robust FGF signaling is not required for cells to populate these tissues. We conclude that ventral foregut endoderm progenitors that cannot receive an FGF signal are less likely to contribute to lung and liver buds.

### Prolonged FGF signaling is required for lung and liver induction

**Figure 5:**
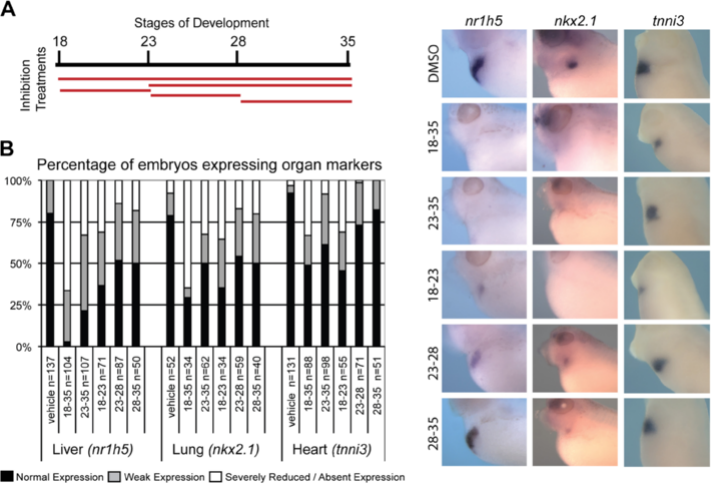
**Prolonged FGF signaling is required for lung and liver induction.** (**A**) Experimental design showing the time-course of FGFRi treatment. (**B**) Embryos treated with DMSO or FGFRi at the indicated stages were assayed at stage NF35 by *in situ* hybridization for markers of liver (*nr1h5*), lung (*nkx2.1*), and heart (*tnni3*). Representative examples are shown and the graph summarizes the % of embryos with normal, weak, or severely reduced/absent expression.

*In vitro* mouse explant experiments using high doses of recombinant FGF signaling induce *Nkx2.1* expressing lung tissue, whereas moderate doses of FGF induce liver-specific gene expression [8]. To test whether *Xenopus* liver and lung fate exhibit a similar dose-dependent need for FGF signaling *in vivo* we treated embryos with different concentrations of the FGFRi from stages NF18 to NF35. This resulted in the expected dose-dependent reduction in endogenous pErk (data not shown). However, both liver (*nr1h5*) and lung (*nkx2.1*) genes exhibited a similar dose responsive reduction in expression (Additional file 2: Figure S2B), suggesting that the level of FGFR activity *per se* does not regulate lung versus liver fate in *Xenopus*. We therefore considered the alternative hypothesis that FGF signaling might induce the liver and lungs at different times in development. To test these temporal requirements for FGF signaling, we treated embryos for different time periods with the FGF receptor inhibitor (Figure 5A) and scored liver and lung specification at stage NF35 (Figure 5B). 

We found that inhibition of FGF signaling for shorter periods of time (NF18-23, NF23-28, and NF23-25) all resulted in a reduction in liver and lung marker expression, which was less dramatic than when FGF signaling was inhibited for the entire NF18-35 period (Figure 5B). We noticed that the level of reduction in *nkx2.1*-expressing lung tissue was similar in the various intermediate treatments, whereas the reduction in liver gene expression was more dramatic at earlier time points. These results are consistent with the tissue separation experiments (Figure 1), which indicate that the liver is specified earlier than the lung. Cardiac *tnni3* expression was mildly reduced across FGF inhibition treatments, with the long and early periods showing the most effects (Figure 5B, Additional file 3: Figure S3A) [50]. These data suggest that prolonged FGF signaling throughout the organ induction period is necessary to specify both liver and lung, and that lung fate requires the longest duration of active FGF signaling.

### PI3K and MEK branches both contribute to lung and liver development

**Figure 6:**
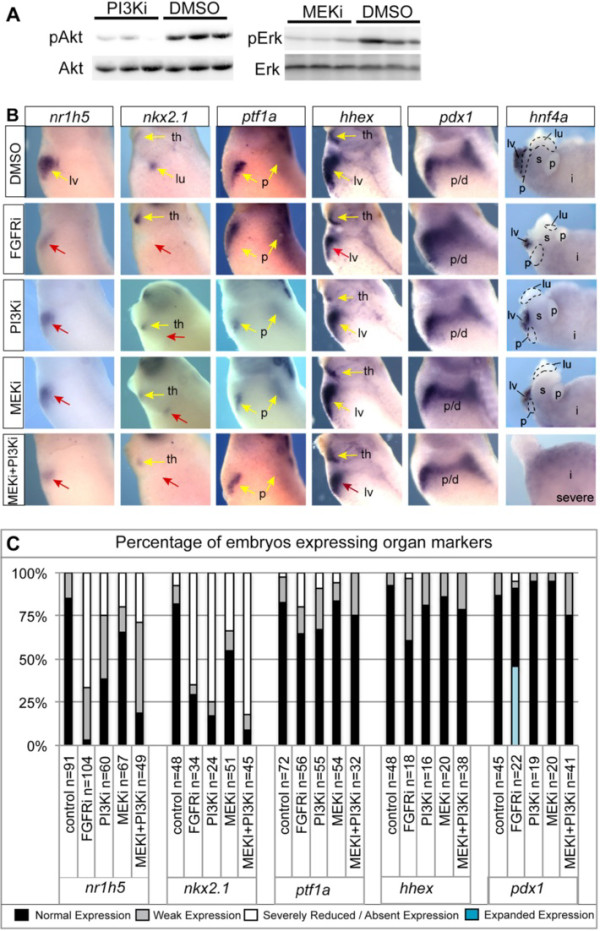
**Both PI3K and MEK signaling contribute to lung and liver development.** (**A**) Western blot analysis of embryos cultured in DMSO, PI3Ki (LY294002), or MEKi (U0126) from stages NF18 to NF35 in triplicate shows a dramatic decrease in pAkt and pErk levels with PI3Ki and MEKi treatment respectively. (**B**) Embryos cultured in DMSO or the indicated inhibitors from NF18 to NF35 were analyzed at stage NF35 for liver (*nr1h5)*, lung (*nkx2.1*), pancreas (*ptf1*α), liver/thyroid (*hhex*), and pancreatic/duodenal (*pdx1*) expression. Embryos cultured in DMSO or the indicated inhibitors from NF18 to NF35 were analyzed at stage NF42 for organ bud appearance with *hnf4*α-stained gut tubes. Yellow arrows indicate normal expression, red arrows indicate reduced or absent expression. Foregut organ buds are outlined in dashed lines (lu; lung, p; pancreas, lv; liver, s; stomach, i; intestine, th; thyroid, p/d; pancreas/duodenum). (**C**) Summary of the percentage of inhibited embryos with expanded, normal, weak, or severely reduced/absent foregut marker expression compared to controls with the number of embryos analyzed listed for each condition. FGFRi data is repeated from Figure 3 for comparison.

There is evidence from mouse foregut explants that different branches of FGF signaling play distinct roles in foregut development, with MEK being required for liver gene expression whist the PI3K branch regulates proliferation [9]. To determine if a distinct FGF signaling pathways were required for foregut lineages in *Xenopus*, we cultured the embryos in either LY294002 (a PI3K inhibitor) or U0126 (a MEK1/2 inhibitor) [53,54] from stage NF18 to NF35 and analyzed the foregut organ lineage markers. Western blot analysis confirmed that PI3K inhibition (PI3Ki) resulted in a significant decrease in phospho-Akt (pAkt), whereas MEK1/2 inhibition (MEKi) resulted in a significant decrease in pErk compared to vehicle treated controls (Figure 6A). PI3Ki and MEKi treatments caused reduced or absent liver expression in 62% or 34% of the embryos respectively, which was not as dramatic as the reductions caused by FGFRi treatment (Figure 6B,C), suggesting that both the PI3K and MEK branches are both involved in *Xenopus* hepatic induction. *Nkx2.1* expression was reduced or absent in 83% of PI3Ki embryos, similar to FGFRi, but in only 45% of MEKi embryos, suggesting prolonged PI3K activity is particularly important in lung specification (Figure 6B,C). If we removed the FGFRi at NF35 and isolated embryonic gut tubes at stage NF42, 46% of the lung and 75% of the liver buds were dramatically reduced in size, while the pancreas, stomach, and intestine were largely unaffected (Figure 6B). In comparison, the PI3Ki or MEKi treatments only caused modest reductions in foregut organ bud size at NF42, which were not as dramatic as those seen with the FGFRi (Figure 6B). We also tested whether PI3K or MEK were required at different times in development by treating embryos for shorter time periods. Similar to the results with the FGFRi, treatment with either MEKi or PI3Ki for a variety of shorter durations between stage NF18-35 all resulted in a modest reduction in lung and liver gene expression which was less severe than the FGFRi treatment over the similar period; and caused little change in pancreas specification consistent with the FGFRi data (Additional file 3: Figure S3A). This data suggests there is not a specific time when the PI3K or MEK branches act, but support a duration model where both branches are necessary over a prolonged period (NF18-35). 

The fact that PI3Ki and MEKi both resulted in moderate phenotypes less severe than FGFRi treatment suggests that PI3K and MEK branches act in parallel. To directly test the dual need for PI3K and MEK signaling, we treated embryos with both inhibitors and observed a dramatic loss of liver and lung markers similar to FGFRi treatment (Figure 6B,C). Additionally, although many MEKi + PI3Ki embryos died by stage NF42, some remaining embryos exhibited severe foregut organ agenesis (Figure 6B). Treatment with PI3Ki, MEKi, or MEKi + PI3Ki did not disrupt expression of *hhex* or *pdx1* in the embryos at stage NF35 (Figure 6B,C), with the exception of a modest decrease in the *hhex* expression area in PI3Ki treated embryos similar to FGFRi (Additional file 3: Figure S3B). The observation that combined MEK + PI3Ki treatment did not expand *pdx1* expression like the FGFRi embryos suggests either that another pathway downstream of FGFR represses pancreatic fate, and/or that PI3K and MEK have additional FGF-independent pro-pancreatic roles in the foregut (Figure 6B,C, Additional file 3: Figure S3B).

We therefore examined the impact that FGF, PI3K, and MEK inhibition had on proliferation and cell death. Embryos treated with FGFRi, PI3Ki, or MEKi did not exhibit significantly changed foregut cell proliferation at stages NF23 or NF35 relative to vehicle treated controls as measured by phospho-histone H3 (pHH3) (Additional file 4: Figure S4A, B). However, at stage NF42, when organ buds undergo extensive outgrowth [55], FGFRi and PI3Ki embryos exhibited a significant decrease in foregut cell proliferation (Additional file 4: Figure S4A,B). Analysis of apoptosis by activated caspase-3 immunostaining demonstrated that PI3Ki (but not FGFRi or MEKi) treatment increased foregut endoderm cell death at stage NF23 and NF35 (Additional file 4: Figure S4C,D). The fact that PI3Ki causes increased cell death, whereas the FGFRi did not supports the idea that additional FGF-independent PI3K-mediated signaling pathway(s) promote foregut cell survival.

We conclude that both the PI3K and MEK branches of FGF signaling contribute to lung and liver specification in *Xenopus* with the PI3K branch being particularly important for lung. In addition FGFR and PI3K regulate organ bud proliferation with FGF-independent PI3K activity also promoting *Xenopus* foregut cell survival.

## Discussion

The role of FGF signaling in *Xenopus* foregut organ development has not been systematically examined to date. In this study we show that:

1. The pancreas, liver and lung are specified at progressively later times in development and that the liver and lung require progressively longer mesodermal contact between stages NF16-35;

2. Activated pErk, indicative of FGF signaling, is detected in the developing foregut endoderm throughout this period of development;

3. Prolonged FGF signaling via both the MEK and PI3K pathways is required for *Xenopus* lung and liver specification *in vivo* and this is at least in part a cell autonomous requirement in the endoderm;

4. FGF signaling regulates the allocation of *hhex* + liver progenitors versus *pdx1*+ pancreatic/duodenum progenitors.

Horb and Slack have previously shown that contact with the mesoderm between stages NF15-42 was essential for the endoderm to express liver and pancreas genes at stage NF42 [26]. Our work extends this study by examining if different lineages are specified at different time points during development and tests the role of FGF signaling in this process. Our data show that the liver and lung lineages are specified at progressively later times in development, requiring mesodermal contact for increasingly longer times. The critical period of mesodermal contact between stages NF16-35 correlates with the presence of pErk in the foregut endoderm and the necessity for prolonged FGF signaling for the expression of liver and lung, but not pancreas, specification markers. We demonstrated that specification of liver and lung lineages requires mesoderm contact from stages NF16-31 and NF16-35 respectively. Interestingly, we found that as early as stage NF16-19 endoderm explants without mesoderm were able to express pancreatic markers *pdx1* and *ptf1a* at stage 35. This result contrasts with those of Horb and Slack who found that the mesoderm was required between stages NF25-42 for anterior endoderm to express *pdx1* at stage NF42 when organ buds begin to form. This suggests that although we detect *pdx1* expression in endoderm explants cultured without mesoderm at stage NF35, pancreatic fate might not yet be stably committed; thus mesodermal contact could be required through stage NF42 to maintain later pancreatic gene expression. This is consistent with the role of FGF/PI3K-mediated signaling promoting proliferation of a *pdx1*+ cell population.

Our data suggest that the prolonged requirement for the mesoderm in liver and lung specification can, at least in part, be accounted for by prolonged FGF signaling. Together with the recent report by Drysdale and colleagues who similarly found that FGF signaling was required for *Xenopus* lung development [46], our data demonstrate that the critical role for FGF signaling in liver and lung is highly conserved in vertebrates [3,6-10,56]. Our results are consistent with findings from chick [7,57] and mouse [8,10,12] explant cultures showing that mesodermal contact and FGF signaling are required for the specification of liver and lung. In addition, although the lateral plate mesoderm is clearly patterned by FGFs [50], our results targeting the dnFGFR to the foregut endoderm demonstrates a cell autonomous requirement for active FGF signaling in liver and lung development similar to endoderm-specific transgenic analysis in mice [9]. It is worth noting that while the molecular pathways are conserved between different species the relative timing of lineage specification is somewhat different. We show that liver and lung fate is specified in *Xenopus* between the 30–37 somite stages (ss), which is similar to zebrafish at 26–30 ss [58]; however in mouse and chick these fates are specified between the 7–10 ss [8,59]. We speculate that these differences could be influenced in part by the relatively early heart development in amniotes.

*In vitro* mouse foregut explants studies suggest that different doses of FGF segregate the foregut endoderm into pancreas, liver and lung lineages with little or no FGF being required for pancreas, intermediate FGF doses promoting liver and high FGF concentration promoting lung fate in the foregut explants [8]. Similarly *hhex* expression in hepatoblasts is FGF-dependent in zebrafish embryos [6] and chick explants [7]. Our results are in general agreement with these studies; we also found that FGF signaling promoted liver and lung and repressed pancreas. However we did not find any evidence supporting a dose requirement for liver versus lung induction in *Xenopus*, although we cannot totally rule out this mechanism. We also did not find discrete short periods in development when liver versus lung fate was induced. Rather our data support a duration model where prolonged FGF signaling is required to specify both liver and lung lineages, with the lung seeming to require a longer duration of FGF activity. Different durations of FGF signaling are important in the development of other systems as well, for instance, in lens epithelial cells it has been shown that low doses of FGF signaling (associated with a short duration of active pErk signaling) promote proliferation while high doses of FGF (associated with prolonged pErk signaling) promote fiber differentiation [60].

We also postulate that the spatial requirement for FGF signaling *in vivo* is also likely to be important factor in regulating the duration of exposure to a FGF signal. We observed that in some FGF inhibited embryos the residual liver gene (*nr1h5*) expression was immediately next to the cardiac mesoderm, suggesting liver gene expression was only induced in the cells closest to a source of FGF. It is important to point out that our experiments do not formally demonstrate that the mesoderm alone is the source of the FGF ligands; evidence from chick and *Xenopus* embryos shows the endoderm also expresses FGF ligands [7,52] and thus autocrine signaling within the endoderm could be involved.

Our data indicate that both the MEK and PI3K branches of the FGF response contribute to lung and liver induction. Inhibition of either branch resulted in intermediate phenotypes compared to FGFRi, while combining MEKi and PI3Ki caused a dramatic loss of mature liver and lung markers recapitulating the FGFRi treatment. Our data also indicate that the PI3K activity is important for cell survival and proliferation, and some of this activity appears to be FGF-independent, consistent with a report that pAkt signaling has an anti-apoptotic role in *Xenopus* stomach/pancreas development [29]. Our *in vivo* findings that both MEK and PI3K are involved in liver and lung specification differ somewhat from explant studies of the mouse liver, where the MEK branch is important for hepatic gene expression while the PI3K branch is important for explant growth [9].

We demonstrated that specification of liver and lung lineages requires mesoderm contact from stages NF16-31 and NF16-35 respectively and while our data suggest that prolonged FGF signaling accounts for this in part, it is likely that additional signals also differentially promote these lineages at later stages. Indeed BMP, Wnt and Retinoic acid are also required for foregut organogenesis [6,7,11,25,48,61,62] and these probably interact with the FGF pathway. Recent examples of such signaling cross talk have come from studies in zebrafish suggest that FGF signaling acts along the anterior-posterior axis to restrict the competence of the endoderm to respond to hepatic-inducing Wnt signals [13,14]. Consistent with other signaling factors acting in concert with FGFs, we found that exogenous FGF2 was not sufficient to support liver or lung fate in foregut explants lacking mesoderm, whereas *in vivo*, and presumably in the presence of other signals, activated caFGFR was sufficient to expand liver and lung at the expense of pancreas. This suggests either that different FGF ligands are specifically required for liver and lung induction and/or that other mesodermal signals are required to potentiate the inducing activity of FGF2. Candidates for additional signals include BMPs. It is also possible that the mesoderm provides important cell-cell or cell-ECM contacts that might be necessary to support foregut organ cell fate. Indeed, we have recently shown that fibronectin-dependent BMP signaling is required to maintain foregut progenitors [48]. In the future it will be important to better understand the mechanisms by which the FGF pathway interacts with other pathways during foregut organogenesis.

## Conclusions

The *Xenopus* embryo is increasingly being used to study the development of endoderm derived organs, but the molecular basis foregut lineage specification is poorly understood. We demonstrate that the liver and lung lineages are specified at progressively later times in development requiring progressively longer mesoderm contact between stages NF15-35. We show that FGF signaling is active in the foregut endoderm at this time and that lung and liver induction requires prolonged FGF signaling through both the MEK and PI3K transduction pathways. We conclude that FGF-mediated foregut organ development in *Xenopus* is highly conserved with that described in mammals. Moreover our results highlight a previously unappreciated role for the temporal regulation of signaling during organ induction, which may impact strategies to direct the differentiation of stem cells.

## Abbreviations

FGF: Fibroblast growth factor; FGFR: Fibroblast growth factor receptor; ERK: Extracellular-signal regulated kinase; pErk: Phosphorylated extracellular-signal regulated kinase; AKT: Protein kinase B; pAkt: Phosphorylated protein kinase B; MEK: Mitogen-activated protein kinase; PI3K: Phosphoinositide 3-kinase; BMP: Bone morphogenetic protein; NF: Nieuwkoop and faber stage; FGFRi: Fibroblast growth factor receptor inhibitor (PD173074); PI3Ki: Phosphoinositide 3-kinase inhibitor (LY294002); MEKi: Mitogen-activated protein kinase inhibitor (U0126); dnFGFR: Dominant negative FGF receptor; caFGFR: Inducible constitutively active FGF receptor; ss: Somite stage.

## Competing interests

The authors declare that they have no competing interests.

## Authors’ contributions

ETS performed most of the experiments and co-wrote the paper. APK performed the explant experiments and co-wrote the paper. SAR participated in the experimental design, performed some of the *in situ* analysis and co-wrote the paper. AMZ guided the project, helped design the experiments and co-wrote the paper. All authors read and approved the final manuscript.

## Supplementary Material

Additional file 1 **Figure S1.** Exogenous FGF is not sufficient to induce lung or liver lineages in explants. (A) Foregut explants with or without mesoderm were cultured from stage NF18 to NF35 in BSA or FGF2 and analyzed for expression of liver (*nr1h5*), lung (*nkx2.1*) and pancreas (*pdx1*) markers. (B) Western blot analysis of explants shows an increase in pErk levels upon FGF2 treatment. Click here for file

Additional file 2 **Figure S2.** FGF signaling is not required for maintaining foregut progenitors, but is required for lung and liver induction in a dose-dependent manner. (A) Embryos cultured in DMSO and FGFRi from stages NF15 to NF23 or injected with RNA encoding β-gal (3 ng) or dnFGFR (3 ng) were analyzed for expression of the foregut progenitor marker *hhex* and the cardiac progenitor marker *nkx2.5*. The graph summarizes the percentage of embryos with normal, weak or severely reduced/absent expression. (B) Embryos cultured from stages NF18 to NF35 in DMSO or FGFRi at the indicated concentrations and the percent of embryos with normal, weak, or severely reduced/absent *nr1h5* and *nkx2.1* expression was scored at stage NF35. Click here for file

Additional file 3 **Figure S3.** Prolonged MEK and PI3K signaling are required for a full lung and liver induction. (A) Percentage of embryos treated with either; DMSO, FGFRi, PI3Ki or MEKi for the indicated stages that exhibit with normal expression of *nr1h5*, *nkx2.1*, ptf1α, or *tnni3* (n > 14 embryos for each condition and probe). Inhibition of MEK or PI3K over various intermediate durations results in a reduction in specification markers, suggesting that prolonged signaling is required for full foregut organ gene expression. (B) Quantification of average *hhex* and *pdx1* expression areas in control and inhibited embryos. ImageJ software was used to measure the expression area with the average expression area of controls set to 1. Averages are based on n > 13 embryos for each condition and marker from at least two independent experiments, standard deviation and significance based on *t*-test (**p < .004) as indicated. FGFRi data is repeated from Figure 3 for comparison. Click here for file

Additional file 4 **Figure S4.** Cell Proliferation and apoptosis in FGF-inhibited embryos. (A) Analysis of cell proliferation by phospho-histone H3 (pHH3) immunostaining in embryos treated with DMSO, FGFRi, PI3Ki or MEKi. At stages NF23 (mid-sagittal) and NF35 (transverse section) embryos were assayed by 80 μM confocal Z-stack of pHH3 immunostaining (white dots), whereas stage NF42 isolated gut tubes were assayed by pHH3 immunohistochemistry (blue dots). Yellow dashed lines outline the foregut region quantified. (B) Summary of mean number of pHH3+ cells in the foregut region +/− SD (n > 5 embryos for each condition and stage). (C) Analysis of apoptosis by activated Caspase-3 immunostaining in embryos treated with DMSO, FGFRi, PI3Ki or MEKi. At stages NF23 (mid-sagittal) and NF35 (transverse section) embryos were assayed by 80 μM confocal Z-stack immunostaining (white dots). Yellow dashed lines outline the foregut region quantified. (D) Summary of mean number of active caspase-3+ cells in the foregut region +/− SD (n > 5 embryos for each condition and stage). Click here for file
