# Supplementary figures and images for: Prolonged FGF signaling is necessary for lung and liver induction in Xenopus
Source: BMC Dev Biol. 2012 Sep 18;12:27. doi: 10.1186/1471-213X-12-27 (PMC3514138; doi:10.1186/1471-213X-12-27)

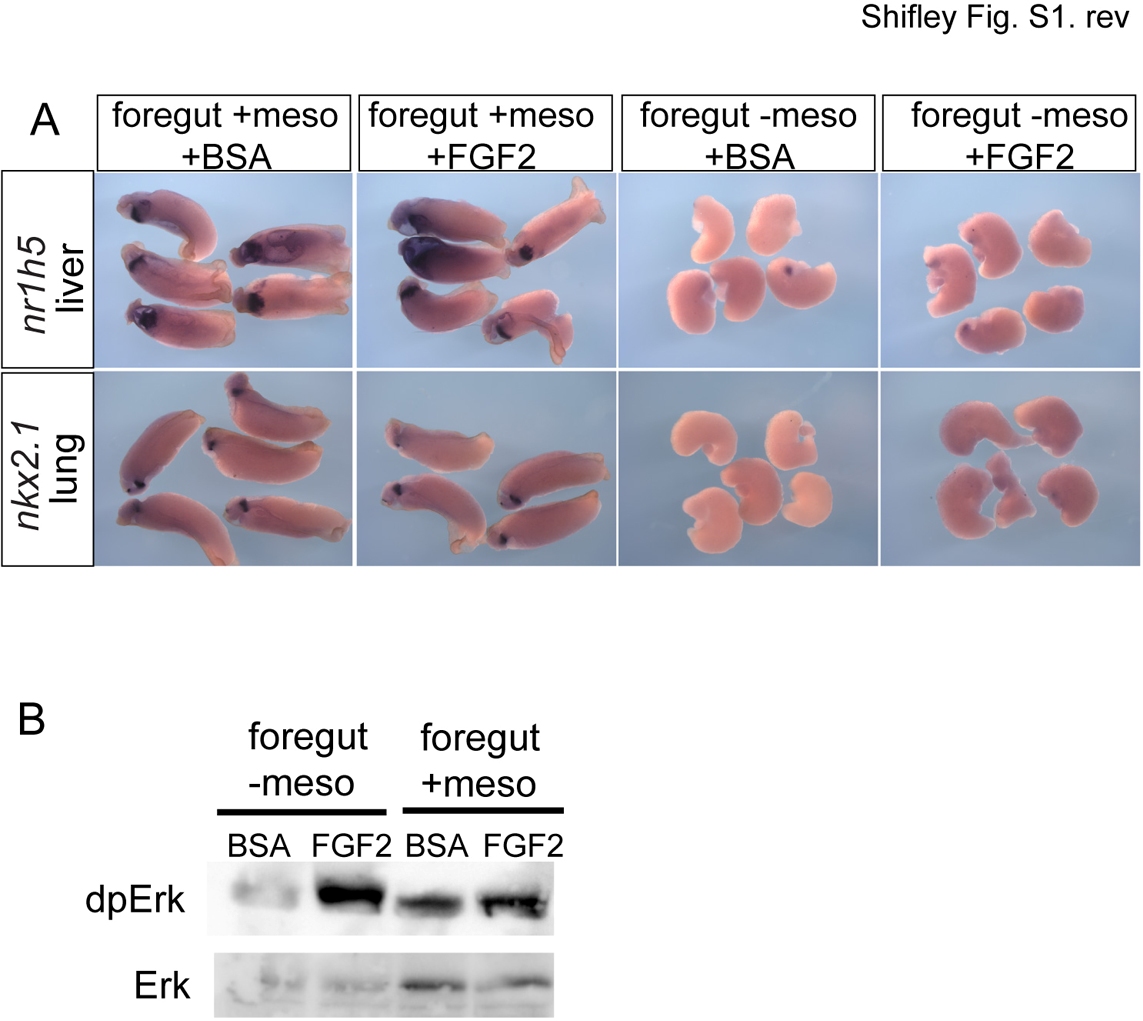

Supplement: Additional file 1 — Figure S1. Exogenous FGF is not sufficient to induce lung or liver lineages in explants. (A) Foregut explants with or without mesoderm were cultured from stage NF18 to NF35 in BSA or FGF2 and analyzed for expression of liver (nr1h5), lung (nkx2.1) and pancreas (pdx1) markers. (B) Western blot analysis of explants shows an increase in pErk levels upon FGF2 treatment. [file 1471-213X-12-27-S1.jpeg]

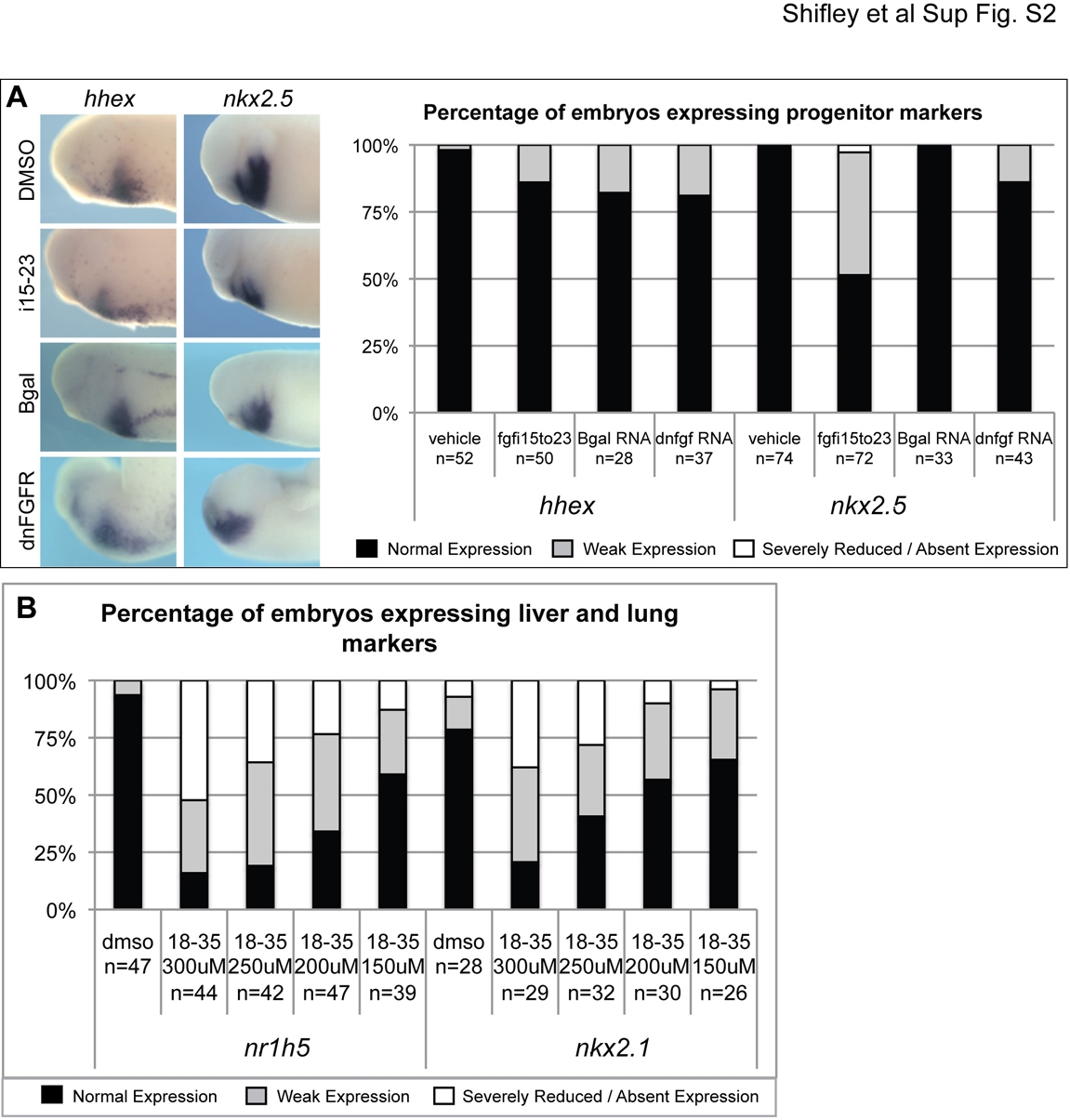

Supplement: Additional file 2 — Figure S2. FGF signaling is not required for maintaining foregut progenitors, but is required for lung and liver induction in a dose-dependent manner. (A) Embryos cultured in DMSO and FGFRi from stages NF15 to NF23 or injected with RNA encoding β-gal (3 ng) or dnFGFR (3 ng) were analyzed for expression of the foregut progenitor marker hhex and the cardiac progenitor marker nkx2.5. The graph summarizes the percentage of embryos with normal, weak or severely reduced/absent expression. (B) Embryos cultured from stages NF18 to NF35 in DMSO or FGFRi at the indicated concentrations and the percent of embryos with normal, weak, or severely reduced/absent nr1h5 and nkx2.1 expression was scored at stage NF35. [file 1471-213X-12-27-S2.jpeg]

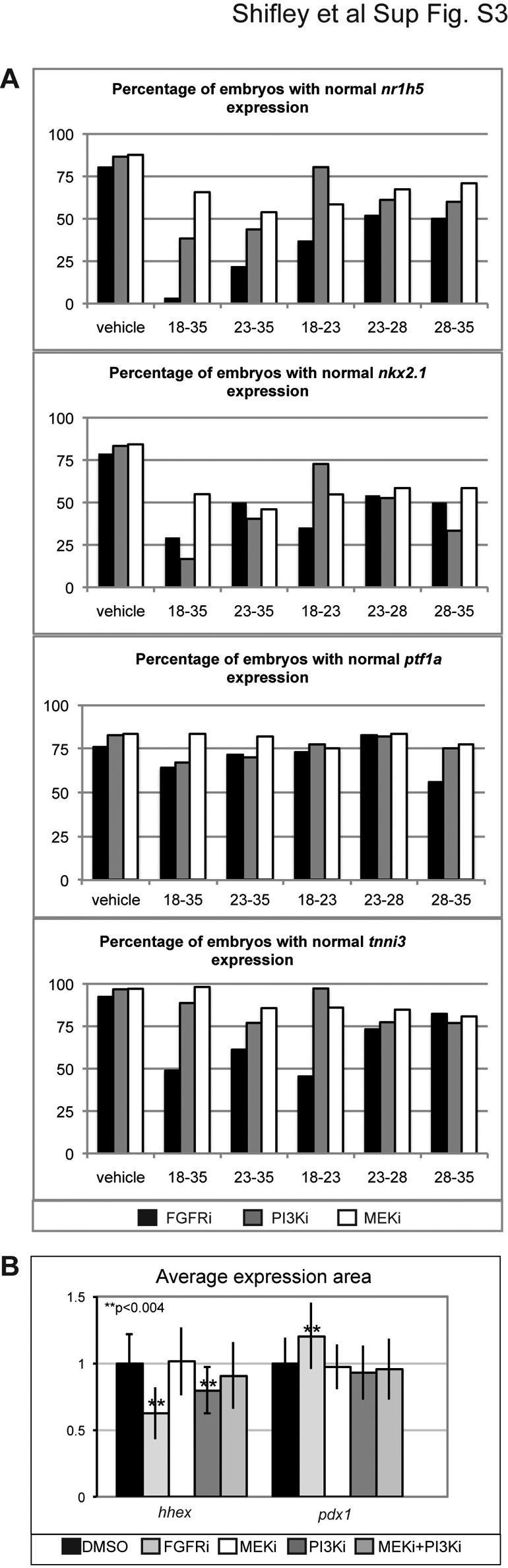

Supplement: Additional file 3 — Figure S3. Prolonged MEK and PI3K signaling are required for a full lung and liver induction. (A) Percentage of embryos treated with either; DMSO, FGFRi, PI3Ki or MEKi for the indicated stages that exhibit with normal expression of nr1h5, nkx2.1, ptf1α, or tnni3 (n > 14 embryos for each condition and probe). Inhibition of MEK or PI3K over various intermediate durations results in a reduction in specification markers, suggesting that prolonged signaling is required for full foregut organ gene expression. (B) Quantification of average hhex and pdx1 expression areas in control and inhibited embryos. ImageJ software was used to measure the expression area with the average expression area of controls set to 1. Averages are based on n > 13 embryos for each condition and marker from at least two independent experiments, standard deviation and significance based on t-test (**p < .004) as indicated. FGFRi data is repeated from Figure 3 for comparison. [file 1471-213X-12-27-S3.jpeg]

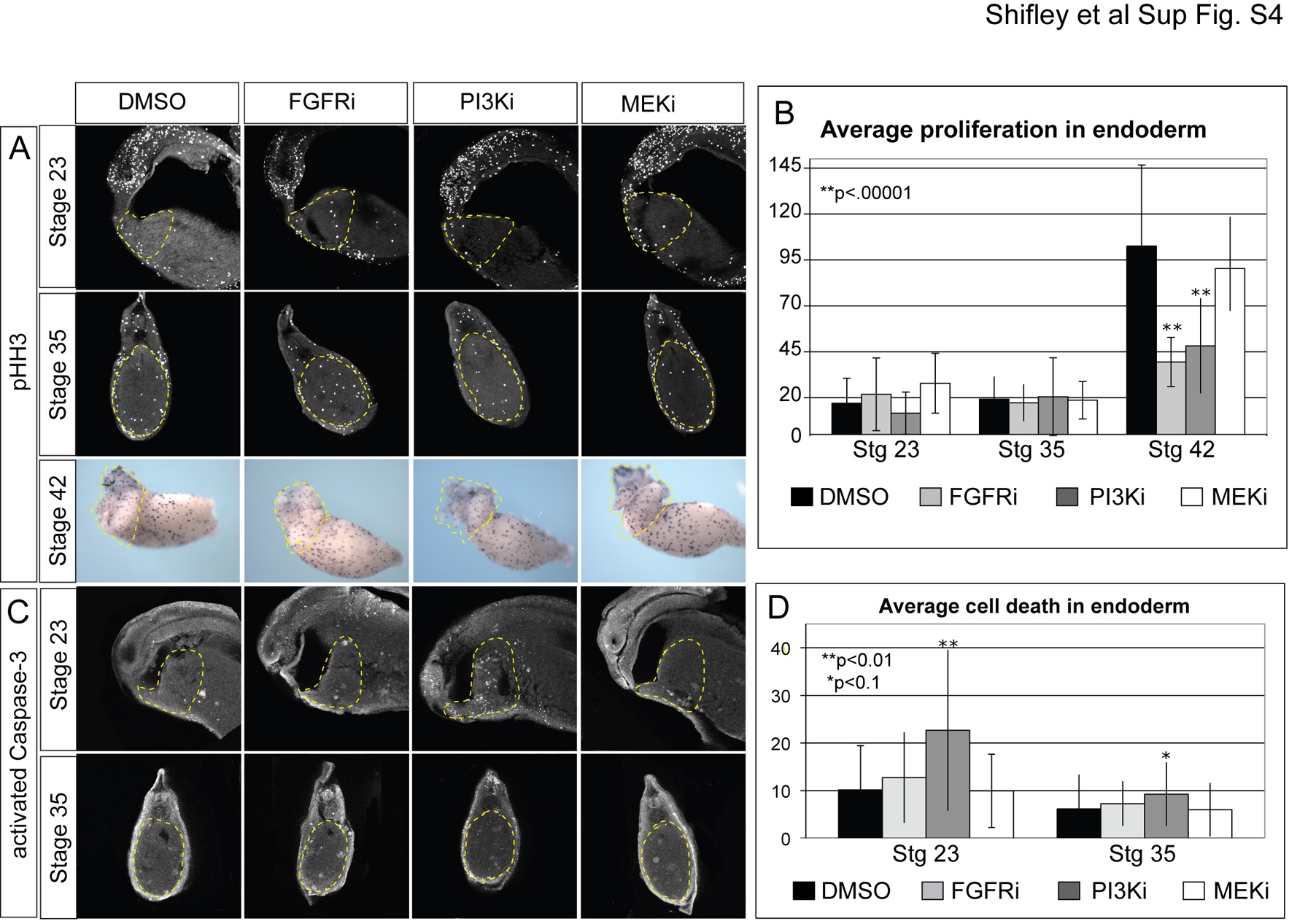

Supplement: Additional file 4 — Figure S4. Cell Proliferation and apoptosis in FGF-inhibited embryos. (A) Analysis of cell proliferation by phospho-histone H3 (pHH3) immunostaining in embryos treated with DMSO, FGFRi, PI3Ki or MEKi. At stages NF23 (mid-sagittal) and NF35 (transverse section) embryos were assayed by 80 μM confocal Z-stack of pHH3 immunostaining (white dots), whereas stage NF42 isolated gut tubes were assayed by pHH3 immunohistochemistry (blue dots). Yellow dashed lines outline the foregut region quantified. (B) Summary of mean number of pHH3+ cells in the foregut region +/− SD (n > 5 embryos for each condition and stage). (C) Analysis of apoptosis by activated Caspase-3 immunostaining in embryos treated with DMSO, FGFRi, PI3Ki or MEKi. At stages NF23 (mid-sagittal) and NF35 (transverse section) embryos were assayed by 80 μM confocal Z-stack immunostaining (white dots). Yellow dashed lines outline the foregut region quantified. (D) Summary of mean number of active caspase-3+ cells in the foregut region +/− SD (n > 5 embryos for each condition and stage). [file 1471-213X-12-27-S4.jpeg]
